# Reading Motivation, Alcohol and Drug Use in Future Teachers in Preschool and Primary School

**DOI:** 10.3390/ejihpe10040080

**Published:** 2020-12-18

**Authors:** María de la Rocha Díaz, Inmaculada Méndez, Cecilia Ruiz-Esteban

**Affiliations:** Department of Evolutionary, Developmental and Educational Psychology, University of Murcia, 30100 Murcia, Spain; mariarocha@um.es (M.d.l.R.D.); cruiz@um.es (C.R.-E.)

**Keywords:** reading, education, higher education, motivation, drugs, alcohol

## Abstract

Future teachers will have to develop the reading habit in their students, as this is an essential factor in schoolchildren. The lack of reading motivation among young people and the need to have it in order to transmit it has been evidenced. Young people often prefer to spend their leisure time using alcohol and other drugs rather than reading books for pleasure. The factors that influence reading motivation are varied, but the objective of this research work focuses on establishing the relationship between reading motivation and the problematic use of alcohol and other drugs in future teachers of Preschool and Primary Education. A total of 178 subjects among university students were recruited (56.6% girls). The ages ranged from 18 to 34 (*M* = 21.59, *SD* = 3.52). The first scale used was the MULTICAGE CAD-4 for behavioral addiction together with a Scale for Characterizing Motivation for Academic Reading (EMLA). The results of the study indicate that those young people who were more involved in the consumption of alcohol and drugs had a lower reading habit. Likewise, the study also reveals significant mean differences in reading motivation based on gender and age. This shows the need to enact healthy habits from the university related to increasing reading motivation and promoting the reading habit in future teachers.

## 1. Introduction

Reading is an essential factor, not only for the acquisition of new knowledge, but also for the development of people’s intellectual capacities. It should be noted that in this article, when talking about reading, we differentiate between leisure reading, that is aimed at enjoyment and acting as a means of entertainment, and merely instrumental reading, that has a didactic or educational function and whose purpose is to obtain knowledge. Likewise, we distinguish between literary reading and digital reading. In this sense, the development of new communication technologies and the multiplication of ways through which new generations access information make young people read daily, but the reality is that such practices occur in a scenario that has nothing to do with reading, this term being understood in a conventional way and irremediably linked to the paper format [1,2,3,4]. Thus, we hereby do not refer to digital reading, since it is not the type of reading that future teachers should promote, but to literary reading [1,5], and more so considering that the digital aspect changes the relationship with the book and therefore does not seem to bring reading closer to those who do not have a taste for it [6].

The importance of reading in education is reflected, for example, in Spain, in article 9.2 of Royal Decree 126/2014 [7], in Primary Education, which establishes an hour of daily reading to encourage reading. This is the reason why future teachers of Preschool Education and Primary Education will necessarily have to promote the development of reading habits in their teaching practice, since the promotion of reading has become an essential activity in the classroom [1,5,8,9,10,11]. 

Given the prominence that reading encouragement currently has in educational centers and the evidence that literary education needs reading mediators capable of transmitting their own personal (and passionate) relationship with books and literature [12], it is essential to know the relationship between teachers and reading in their personal sphere in order to establish ways to guide and motivate future teachers in order to improve teaching practices [13].

The problem arises from the evidence that university students read books and other materials with specific objectives, but not for pleasure [9,10,14,15]. This can be a serious detriment to the quality of teaching for future teachers, since a teacher who maintains a weak relationship with reading cannot help develop intrinsic motivation for reading practice among his students [8,16]. Thus, in the case of university degrees aimed at teaching, as in the case of the Primary Education and Early Years Education degrees, it is necessary to establish the reading motivation of future teachers [10,17] vis-à-vis their future teaching work [13,18]. In this sense, the existence of deficiencies in literary reading training in the teaching curriculum for teachers has been confirmed, which is having certain repercussions on the reading training of Spanish university students [13].

To make this evidence of lack of motivation and interest in reading even greater, certain studies have detected, in addition to a poor reading habit, worrying percentages of future teachers who openly express their disaffection towards reading and the merely instrumental use they make of reading as students [9]. In this way, the promotion of motivation and the development of a reading habit become fundamental in the training of future teachers [10,17], and it is at the university level that these aspects should be promoted in order to achieve motivated teachers capable of instilling the necessary reading habit in their students, since the enthusiasm of a teacher can have positive consequences in the development of the love for reading [12,19,20]. The reading habit is influenced by several internal factors such as age and gender, as well as external factors such as peers, schools, teachers, and library services available to individuals [21,22,23]. In this respect, studies have shown a slight tendency of improvement in their taste for reading and their attitude towards reading as they progress in university studies [3,11], this being greater among working teachers [18].

On the other hand, existing data in our country indicate that those students who do not read books for fun during leisure time tend to make greater use of alcohol and drug use [24]. Whether these are legal drugs, such as alcohol and tobacco, or illegal drugs, such as cannabis, cocaine, or designer drugs. It is estimated that, among young university students in our country, this statement is true for one fifth of the population of this collective [25]. In this sense, it should be noted that the consumption of drugs is a growing problem in young population, especially excessive alcohol use [26]. 

Drugs have begun to play a central role in the life of young people, as they play a facilitating role when facing aversive situations in which they have little sense of control and personal effectiveness [27]. It should be noted that problematic alcohol and drug use has serious repercussions on a comprehensive level for the individual. Specifically, it could have a negative impact at an academic level, for example, on student performance, since problematic alcohol and drug use affects concentration, attention, and memory [28]. Taking these ideas into account, the need to know if future preschool and primary school teachers have a motivation towards reading for pleasure in their free time or if other risky actions appear, such as the consumption of alcohol and other drugs. For all of the above, the objective of this study was to analyze the relationship between the reading motivation among future teachers of Preschool and Primary Education and their consumption of alcohol and other drugs. The second objective was to analyze whether certain sociodemographic characteristics (gender and age) made a difference in terms of reading motivation and the use of alcohol and other drugs.

## 2. Materials and Methods 

### 2.1. Design and Participants 

A cross-sectional design was followed for this study. First, a university center was selected from the southeast of Spain. The Bachelor’s Degree in Early Years Education and the Bachelor’s Degree in Primary were selected. A total of 178 university students were recruited. The participants were aged from 18 to 34 years *(M = 21.59, SD = 3.52*), 63.5% pertaining to studies of primary education and the rest to preschool education. This is a representative sample of young university students studying for the Bachelor’s Degree in Early Years Education and the Bachelor’s Degree in Primary Education from a university located in the southeast of Spain, showing a confidence level of 90% and a margin of error of 5%. As can be seen in Table 1, above all, there were more women among the study participants.

### 2.2. Instruments

The first scale used was the Scale for Characterizing Motivation for Academic Reading (EMLA) [17]. The instrument consists of 27 items ranging from 1 = total disagreement to 6 = total agreement that measure the following factors: expectations, interest (enjoy homework), importance (how relevant it is for the subject to perform a certain task well), usefulness (how well a task fits into the person’s future plans), and cost (how the decision to engage in one activity limits access or the possibility of doing others; that is, how much effort will it take to carry out this activity and what is its emotional cost) (see Table 2 for examples of items). Cronbach’s alpha reliability values are adequate for these subscales: expectations (α = 0.80), interest (α = 0.84), importance (α = 0.90), usefulness (α = 0.83), and cost (α = 0.68) [17]. In our study, it was α = 0.97 for the entire scale.

Second, the questionnaire on behavioral addictions was selected (MULTICAGE CAD-4) [29]. The instrument consists of 24 dichotomous items that analyze the following scales: alcohol abuse/dependence (measures aspects related to the abusive consumption of alcoholic beverages), substance addiction (measures aspects related to problematic drug use), pathological gambling (measures aspects related to gambling addiction), smartphone addiction (measures aspects related to problematic mobile phone use), Internet addiction (measures excessive use of the Internet), and video game addiction (measures aspects related to gambling and use of console or computer games in a problematic way). Cronbach’s alpha reliability for each of the scales ranged between 0.88 and 0.95 in the study original [29]. In our study, it was α = 0.84 for the total scale. For this study, the subscales of alcohol abuse/dependence and substance addiction were selected. See Table 3 for examples of items in each of the subscales.

### 2.3. Procedure

First of all, it was necessary to process the approval by the Ethics Committee of the University of Murcia (ID: 2819/2020). Next, the collaboration of the teachers involved in the different subjects of the Early Years and Primary Education Degrees was processed to request their collaboration to be able to access one of the classes and to be able to administer the assessment instruments. It was necessary to have the informed consent of the participants. In those cases where said consent was not available, these young people were excluded from the study. Participants responded anonymously, voluntarily, and confidentially.

### 2.4. Data Analysis

In this study descriptive techniques such as frequencies, percentages, means, and standard deviations were used. In order to be able to obtain the relationships between the study variables quantitative (between reading motivation and behavioral addictions and Correlations between reading motivation and age), Pearson correlations were performed. Student’s t tests were performed to analyze the differences between the gender based on reading motivation according to the Cohen effect size (d). All analyses were performed using SPSS 24.0 (IBM, Armonk, NY, USA). 

### 2.5. Ethics Approval

The protocol for this study was approved by the Ethics Committee for Clinical Investigations of the University of Murcia in April 2020 (ID: 2819/2020), in accordance with the approved guidelines and with the declaration of Helsinki, obtaining written informed consent forms from all participants. 

## 3. Results

On the one hand, with regard to reading motivation and alcohol consumption, the existence of a significant negative correlation with expectations and usefulness was evidenced, not being significant in the case of interest or cost. 

Regarding drug use, a significant negative correlation was found with all the factors of reading motivation (expectation, interest, importance, usefulness, and cost) (see Table 4). 

Regarding gender differences, in Table 5, we can see that the Student’s t test determined the existence of significant mean differences between reading motivation and gender. Specifically, women’s averages were higher in terms of expectation, interest, usefulness, and cost as compared to men. However, there were no differences in terms of importance.

Regarding age, significant positive correlations were found between expectation, interest, importance, and usefulness with respect to age. However, these were not cost-significant (See Table 6).

## 4. Discussion

Attending to the first objective, we have found that the greater consumption of alcohol and other drugs, the less the reading motivation. This shows that young people with higher reading motivation rates are less involved in risky behaviors such as drug use. In addition, expectation and usefulness were also indicators of a lower involvement in alcohol consumption behaviors. This is in line with what is evidenced by the data reported in Spain that indicate that students who do not read books for leisure have higher prevalence of consumption of addictive substances during leisure time [24].

With regard to the second objective of the study, it has been shown that as the age of university students increases, so do the different factors of reading motivation (expectation, interest, importance, and usefulness). This is in line with other studies that have shown that there seems to be a slight tendency for the figures on reading for pleasure to improve throughout the university studies and the fact that attitude towards literature also improves [5,11], especially in the case of working teachers [18]. As regards gender differences, it should be noted that women obtained higher scores in expectation, interest, usefulness, and cost. However, no differences in terms of importance were found, which indicates that it is indifferent in terms of gender. Thus, we have shown as in previous studies that reading habits and motivation are influenced by several internal factors such as age or gender [21,22].

Data from our study indicate that future teachers of preschool and primary education often show little reading motivation [9], as they usually only read for the purpose of passing the exams but not for the pleasure of acquiring knowledge [9,10,14,15]. In this sense, we have shown that, as in previous studies, university students are very skilled at decoding texts, but they require support and follow-up to develop their reading habits [15].

The result of the data collected shows that it is necessary, from the university level, to try to avoid such behaviors by enhancing healthy habits among university students framed in the promotion of reading [12,13,30]. Given that the future preschool and primary teachers, as motivational agents of the reading habit, must develop healthy habits that allow them to create and maintain a taste for reading in their future students [1,5,9,10,11], it is imperative that the university moves towards a comprehensive, solid, and consistent training including the skills that these future professionals have to develop as reading mediators, enhancing their reading habits in terms of both behavior and reading commitment [3,13,15].

In the same way, it would be convenient for the university to play an essential role, not only in the development of reading skills, but also in avoiding the use of drugs by strengthening preventive actions that promote healthy lifestyles among young people [31] and altering the perception of risk and the social acceptance shown by university students towards the use of drugs [32], being therefore relevant the insertion within the curriculum of subjects focused on preventing drug use as well as the development of psychoeducational strategies in the different academic units of university institutions [25,32].

Regarding the limitations of our study, it should be noted that this is a cross-sectional study focused on a single age bracket and, above all, that the questionnaires may be affected by social desirability. We consider that more comparative studies are necessary between university students and active teachers [18], and furthermore, that they measure variables of interest such as reading habits [10], the academic impact, and the development of educational and innovation programs [30], as well as other personal or family aspects that may be influencing reading motivation.

## 5. Conclusions

The results of the study are indicative that university students who make more use of reading for pleasure in their free time tend to show less consumption of alcohol and other drugs. Gender and age are variables that must be taken into account when carrying out preventive or intervention actions in the university environment.

Thus, in the case of those careers aimed at teaching, as in the case of Early Years Education and Primary Education degrees, it is necessary to increase the training strategies so as to increase the reading habit and interests in the future teachers [10], but also those aimed at avoiding the consumption of drugs and other substances that may have a negative impact on any motivational factors.

Finally, it is essential that the university moves towards a comprehensive, solid, and coherent training including the competences that future education professionals must develop as reading mediators, enhancing their reading habit in terms of both behavior and reading engagement [5,13].

## Figures and Tables

**Table 1:** Main sociodemographic characteristics of the study participants.

Variable		Frequency (n = 178)	Percentage (100%)
Age	18–25 years old 26–34 years old	160 18	89.9% 10.1%
Gender	Male	44	24.7%
	Female	134	75.3%
Country of birth	Spain	171	96.1%
	Abroad	7	3.9%

**Table 2:** Examples of items of the instrument for academic reading motivation.

Subscales	Examples of Items
Subscale expectation	*By reading the academic text, I managed to capture the core ideas*
Subscale interest	*I like to read academic texts related to my career*
Subscale importance	*For me, it’s important to read the bibliography before class (when applicable)*
Subscale usefulness	*I find it very useful to understand academic texts*
Subscale cost	*I am able to put other interests* aside and commit myself until I *properly finish* reading *a text*

**Table 3:** Examples of items in the instrument for behavioral addiction.

Subscales	Examples of Items
Subscale alcohol abuse/dependence	*Have you ever thought that you should drink less?*
Subscale substance addiction	*Do you sometimes feel driven to use drugs, even though you have decided not to?*

**Table 4:** Correlations between reading motivation and behavioral addictions.

	Alcohol	Drugs
Expectation	−0.195 *	−0.188 *
Interest	−0.068	−0.161 *
Importance	−0.101	−0.176 *
Usefulness	−0.150 *	−0.226 **
Cost	−0.139	−0.152 *

Note: * p < 0.05, ** p < 0.01.

**Table 5:** Gender differences based on reading motivation.

Variable	Male M (SD)	Female M(SD)	t	p
Expectation	20.27 (5.53)	22.17 (4.95)	−2.145	0.033
Interest	26.16 (8.06)	29.02 (7.40)	−2.180	0.031
Importance	22.14 (6.37)	23.60 (6.47)	−1.311	n.s. ^1^
Usefulness	20.37 (5.51)	22.57 (4.87)	−2.518	0.013
Cost	15.02 (4.49)	17.12 (4.01)	−2.918	0.004

^1^ Note: n.s. not significant.

**Table 6:** Correlations between reading motivation and age.

	Age
Expectation	0.175 *
Interest	0.150 *
Importance	0.157 *
Usefulness	0.146 *
Cost	0.125

Note: * p < 0.05.
